# Knowledge and Attitudes of Dental Professionals in Lithuania Toward Child Abuse and Neglect: A Cross-Sectional Survey

**DOI:** 10.3390/dj14060328

**Published:** 2026-06-01

**Authors:** Julija Narbutaitė, Vilma Brukienė, Eglė-Aida Bendoraitienė, Vilija Andruškevičienė, Aistė Kavaliauskienė, Severina Petrovskytė, Apolinaras Zaborskis

**Affiliations:** 1 Department of Oral Health and Paediatric Dentistry, Faculty of Odontology, Medical Academy, Lithuanian University of Health Sciences, A.Mickevičiaus 9, LT-44307 Kaunas, Lithuania; egle.aida.bendoraitiene@lsmu.lt (E.-A.B.); vilija.andruskeviciene@lsmu.lt (V.A.); severina.petrovskyte@stud.lsmu.lt (S.P.); 2 Institute of Dentistry, Faculty of Medicine, Vilnius University, Universiteto 3, LT-01513 Vilnius, Lithuania; vilma.brukiene@mf.vu.lt; 3 Department of Orthodontics, Faculty of Odontology, Medical Academy, Lithuanian University of Health Sciences, A.Mickevičiaus 9, LT-44307 Kaunas, Lithuania; aiste.kavaliauskiene@lsmu.lt; 4 Department of Preventive Medicine & Health Research Institute, Faculty of Public Health, Medical Academy, Lithuanian University of Health Sciences, A.Mickevičiaus 9, LT-44307 Kaunas, Lithuania; apolinaras.zaborskis@lsmu.lt

**Keywords:** dentists, students, oral health, knowledge, attitudes, children, abuse, neglect

## Abstract

**Background/Objectives**: Dentists, due to their close contact with patients, are in a unique position to recognize and report cases of child abuse and neglect. This study aimed to explore dentists’ knowledge and attitudes toward child abuse and neglect encountered in their professional practice. **Methods**: An anonymous questionnaire survey was conducted among 414 members of the Lithuanian Dental Chamber (general dentists, dental specialists, dental hygienists, and dental assistants) and 153 graduating dental students, yielding a total sample of 567 respondents. They reported knowledge and attitudes regarding reasons for child abuse within the family and barriers to referring suspected cases. The underlying structure of responses was examined using exploratory factor analysis, and differences in knowledge and attitudes across professional groups were tested using ANOVA. **Results**: Most respondents agreed that low family socioeconomic status (87.7%), child’s disability (65.3%), and medical conditions (53.2%) are the main reasons for child abuse. These and other reasons clustered into two factors: family social vulnerability, and child health-related vulnerability; however, professional specialization had only a very small effect on both factors (η^2^ = 0.013 and η^2^ = 0.017, respectively). The majority of respondents (75.6%) agreed that dentists should report cases of child abuse or violence against children in all circumstances. The barriers to reporting child abuse were fear of negative consequences, and professional uncertainty; professional specialization had a significant effect on both of these factors (η^2^ = 0.019, *p* = 0.027 and η^2^ = 0.037, *p* < 0.001, respectively). Dental specialists reported the highest levels of fear of negative consequences and professional uncertainty, whereas students reported the fewest difficulties in reporting. Dental specialists and students demonstrated the highest levels of knowledge, while dental hygienists demonstrated the lowest level of knowledge regarding child abuse (mean sum score of knowledge was 11.2 (SE 0.38) vs. 10.0 (SE 0.30), respectively, *p* = 0.014). **Conclusions**: Overall, Lithuanian dentists, including students, demonstrate moderate knowledge and attitude in recognizing cases of child abuse and neglect; however, differences across professional groups remain and should be addressed to ensure more effective child protection.

## 1. Introduction

Child maltreatment, encompassing physical, psychological, and sexual abuse, as well as neglect, constitutes a major global public health concern with profound and enduring consequences for physical health, mental well-being, social functioning, and overall quality of life across the lifespan [1,2]. Exposure to maltreatment in childhood has been consistently associated with adverse developmental outcomes, including impaired cognitive and emotional development, increased risk of chronic disease, mental health disorders, substance misuse, and reduced educational and socioeconomic attainment. Despite growing awareness, child maltreatment remains widespread and frequently undetected or underreported worldwide [3,4].

According to the World Health Organization (WHO), up to six out of ten children under the age of five are exposed to physical or psychological violence, while approximately one in five women and one in seven men report having experienced sexual abuse during childhood [2]. These figures likely underestimate the true prevalence, as many cases remain hidden due to stigma, fear, and inadequate recognition by professionals. Consequently, healthcare professionals play a pivotal role in the early identification, prevention, and management of child maltreatment.

Dental professionals are uniquely positioned in this regard, as injuries involving the head, face, and oral cavity are among the most frequent physical indicators of abuse [5,6,7,8]. Students, dentists, dental hygienists, and dental assistants regularly examine these body areas and may therefore be among the first professionals to observe clinical signs suggestive of maltreatment. In addition to physical injuries, behavioural indicators such as dental neglect, extreme anxiety, or inconsistent explanations for injuries may further raise suspicion.

However, existing evidence demonstrates that many healthcare professionals lack sufficient knowledge regarding the various forms of child maltreatment, their clinical manifestations, and the legal and ethical responsibilities associated with reporting suspected cases [9,10,11]. Moreover, inadequate personal attitudes and beliefs, uncertainty about diagnosis, and fear of legal consequences or damaging relationships with families may contribute to hesitancy or failure to report suspected abuse.

Research also indicates that inadequate education and training during undergraduate and professional studies can negatively influence future clinical behaviour and confidence in managing suspected child maltreatment cases [12,13,14,15]. Variations in knowledge, attitudes, and reporting practices among different groups of dental professionals underscore the need for structured, targeted, and discipline-specific educational interventions [16,17,18].

In Lithuania, child maltreatment and neglect continue to represent a significant social and public health concern. Official statistics from child protection authorities indicate thousands of reported cases annually; however, research suggests that these figures likely underestimate the true prevalence. Findings of population-based studies and retrospective surveys conducted among adolescents and young adults show that a substantial proportion of individuals report having experienced at least one form of maltreatment during childhood. Psychological violence and neglect appear to be among the most prevalent forms, while physical and sexual abuse are also reported, albeit less frequently in official records [19]. Additionally, research has identified family-related risk factors, such as alcohol misuse, socioeconomic difficulties, and limited access to support services, as commonly associated with cases of child neglect and abuse [20].

Studies conducted in Lithuania further highlight the persistence of social norms that may contribute to the normalization or minimization of certain forms of violence against children. Surveys assessing public attitudes have shown that a notable proportion of adults still perceive physical punishment as an acceptable child-rearing practice. Such attitudes may hinder the recognition of maltreatment and contribute to delayed intervention by both families and professionals [18]. In Lithuania, however, empirical data on the preparedness of healthcare professionals to recognize and respond to child maltreatment remain limited. Available pilot studies suggest variability in knowledge and attitudes across different professional groups, underscoring the need for systematic and interdisciplinary training. Given the limited number of comprehensive studies addressing child maltreatment within the Lithuanian healthcare system, particularly in dentistry, there is a clear need for further research.

In view of the above, this study aimed to explore dentists’ knowledge and attitudes toward child abuse and neglect encountered in their professional practice. The following specific objectives were defined: (i) to explore dentists’ knowledge and attitudes regarding the reasons for child abuse and neglect within the family; (ii) to examine dentists’ attitudes toward reporting suspected cases of child abuse and neglect; (iii) to assess dentists’ knowledge of child abuse and neglect; and (iv) to elaborate future guidelines for child abuse prevention through the use of professional opportunities. Based on these objectives, we tested the hypothesis that knowledge and attitudes differ across dentists’ professional specialization groups, namely dentists (general practitioners), dental specialists, dental hygienists, dental assistants, and graduating dental students, as well as across respondents’ sex and age groups.

## 2. Materials and Methods

### 2.1. Sample Size, Participants, and Ethics

A cross-sectional survey was planned among five groups of respondents involved in dental practice: general dentists, dental specialists, dental hygienists, dental assistants, and dental students. The dental specialist group comprised dentists with an acquired postgraduate qualification (e.g., pediatric dentistry, oral surgery, orthodontics, periodontics, or prosthodontics).

The sample size was estimated using a priori power analysis in G*Power 3.1.9.4 software (University of Düsseldorf, Düsseldorf, Germany) for a one-way ANOVA (*F* test) with five independent groups. Assuming a significance level of α = 0.05, statistical power of 0.80, and a small-to-medium effect size (Cohen’s *f* = 0.15, corresponding to a partial η^2^ = 0.02), the required total sample size was 536 participants, corresponding to at least 100 participants per group.

The inclusion criteria were the provision of informed consent to participate in the survey and completion of the questionnaire. The study sample included members of the Lithuanian Dental Chamber who were employed full- or part-time, without restrictions on age or years of professional experience (the Chamber had approximately 8600 members at the time). Additionally, all graduating dental students from the Lithuanian University of Health Sciences and Vilnius University were invited to participate in the study (a total of 164 students).

The study protocol was approved by the Lithuanian Committee for Biomedical Research Ethics on 9 June 2022 (Decision No. 613-22-96). Data collection was conducted in accordance with the principles of the World Medical Association’s Declaration of Helsinki. Informed consent was obtained from all participants, and their anonymity and confidentiality were fully ensured.

### 2.2. Questionnaire and Variables

Data were collected using a self-administered questionnaire, developed by the study authors based on previous similar studies [12,21,22] and our own professional experience (Supplementary File S1). The face and content validity of the questionnaire was assessed through a pilot survey amongst 10 dental students from the Lithuanian University of Health Sciences. The students were asked to provide feedback over clarity, comprehensibility and relevance of the questionnaire items and response formats, and report any ambiguity. Appropriate modifications were performed in the study questionnaire. However, formal assessment of construct validity and test–retest reliability was beyond the scope of this study. The pilot study participants were excluded from the final analysis. The average time required to complete the questionnaire was approximately 10 min.

The questionnaire consisted of four sections. The first section collected participants’ demographic and professional characteristics, including gender, age, place of residence (urban or rural), years of professional experience, employment sector (public or private), and professional specialization.

The second section comprised questions assessing respondents’ opinions on risk factors and signs of child abuse, as well as their views on appropriate actions when child abuse is suspected. In addition to these items, two scales developed specifically for this study were included.

The first scale, “Reasons for Child Abuse in the Family”, was designed to assess respondents’ perceptions of factors contributing to child abuse, neglect, or violence within the family. It consisted of nine statements describing potential reasons why a child may experience abuse or neglect (e.g., “child with a disability”, “child with a medical condition”, “low socioeconomic status of the family”). Responses were rated on a five-point Likert scale ranging from 1 (“strongly agree”) to 5 (“strongly disagree”). For the statistical analysis, answers were grouped as “strongly agree or agree”, “neither agree nor disagree” and “disagree or strongly disagree”. In the present study, this scale demonstrated good internal consistency (Cronbach’s α = 0.831).

The second scale, “Barriers to Referring Suspected Child Abuse”, was developed to examine dentists’ attitudes toward obstacles to reporting suspected child abuse cases to social services. The scale comprised 15 statements describing potential barriers that could hinder respondents’ efforts to report child abuse (e.g., “fear of negative impact on dental practice”, “fear of family violence toward the child”, “lack of knowledge of referral procedures”). Responses were rated on the same five-point Likert scale (1 = “totally agree” to 5 = “totally disagree”). For the statistical analysis answers were grouped as “strongly agree or agree”, “agree nor disagree” and “disagree or strongly disagree”. In the present study, this scale demonstrated acceptable internal consistency (Cronbach’s α = 0.727).

The third section consisted of questions addressing respondents’ training history in child protection, as well as their views on the need for continuing education in the recognition and reporting of child abuse and neglect. This section also included a set of 15 questions designed to assess respondents’ knowledge of child abuse. Each item was scored as either correct or incorrect, and overall knowledge was evaluated based on the total number of correct answers.

The fourth section presented two clinical case scenarios. For each case, participants were asked to assess whether the scenario represented child abuse or neglect and, if so, to identify the type of abuse or neglect involved. These data were not used in present analyses.

### 2.3. Procedures

In the first phase, a comprehensive theoretical framework for the study was developed through a review of scientific databases. Based on the defined study objectives, the research methodology and study participants were specified. During the development of the questionnaire, the variables under investigation, as well as the instrument’s validation and psychometric properties, were carefully considered. Content validity was assessed through a pilot survey, as described above, and the final version of the instrument was approved by consensus among the study authors.

In the second phase, the research protocol, including the informed consent document and the instruments to be used, was submitted to the Lithuanian Committee of Bioethics for approval. Following a favourable decision, contact was established with the Lithuanian Dental Chamber to request permission to administer a questionnaire survey to its members during national and regional conferences or other professional events. The student survey was coordinated with the faculty administration.

The final phase involved data collection. From 15 October 2022 to 15 June 2023, one national conference and five regional professional conferences (training events) organized by the Lithuanian Dental Chamber were held across the country. At the registration desk, participants were invited to take part in the study by completing a paper-based questionnaire; those who completed the questionnaire were eligible to participate in a lottery. Questionnaires were distributed to participants on a first-come basis at registration, without stratification by professional specialization. Therefore, the invited participants may be considered a convenience subsample. In total, 600 questionnaires were distributed, of which 421 were returned completed. Response rate 70.2%.

The student survey was conducted at the universities during a lecture break. Students were informed about the purpose of the study and the intended use of the collected data. Participation was voluntary; however, all students present in the lecture hall agreed to participate, with completion of the questionnaire indicating informed consent. In total, 153 students participated in the survey, corresponding to a response rate of 93.3% based on the enrolled student lists.

Before the survey, respondents were informed that participation was voluntary, no personal information would be collected, and their anonymity and confidentiality would be fully protected. To protect confidentiality, all data were de-identified, securely stored and accessible only to the investigators.

### 2.4. Data Analysis

After data cleaning, a total of 567 questionnaires, 414 from employed dentists and 153 from students, was included in the analysis. The data were analyzed using a quantitative approach.

First, descriptive statistics were calculated to estimate frequencies (*n*) and percentages (%) for categorical variables, as well as means (*M*), standard deviations (SD), standard errors (SE), and ranges for continuous variables. Bivariate analyses were conducted to examine differences between respondent groups. The chi-square (χ^2^) test and *z*-tests were used to compare proportions of categorical variables, while differences in means were assessed using the General Linear Model (analysis of variance, ANOVA). Within the ANOVA framework, linear regression coefficients (*B*) were estimated to evaluate associations between continuous outcome variables and their predictors. Partial effect size (η^2^) was used to represent the proportion of variance in the dependent variable attributable to a given independent variable after controlling for other variables in the model. Following commonly used guidelines, η^2^ values of approximately 0.01, 0.06, and 0.14 were interpreted as small, medium, and large effects, respectively. ANOVA also allowed adjustment for potential confounders; therefore, both raw means and standardized (marginal) means were used for comparisons.

Before conducting the ANOVA, a sensitivity analysis was performed. This analysis was used to determine whether the outcome variable depended on participants’ sex and age, and therefore whether these variables should be included in the ANOVA model. Sensitivity analysis was not conducted for years of professional experience because this variable was strongly correlated with age. Sensitivity analysis for sex was performed among dentists, dental specialists, and students, as the groups of dental hygienists and assistants consisted exclusively of female participants. Sensitivity analysis for age groups was conducted only among employed participants, since all students belonged to the younger age group. Thus, sex and age were included in the ANOVA model only when the sensitivity analysis indicated their significant association with the outcome variable.

Exploratory factor analysis (EFA) was also used in data processing to identify the underlying structure of the scale items. The suitability of the data for factor analysis was assessed using the Kaiser–Meyer–Olkin (KMO) measure of sampling adequacy (KMO > 0.5) and Bartlett’s test of sphericity (*p* < 0.001). The analysis was conducted using Principal Axis Factoring with direct oblimin rotation, as the items were measured on Likert-type scales and the underlying factors were expected to be correlated. Factor scores derived from the extracted factors were used in subsequent analyses. Although individual items were coded from 1 (totally agree) to 5 (totally disagree), factor scores were recoded so that higher values indicated greater endorsement of the underlying constructs.

All analyses were conducted at *p* < 0.05 level of significance using the SPSS statistical package (version 23.0; SPSS Inc., Chicago, IL, USA). We followed the Strengthening the Reporting of Observational Studies in Epidemiology (STROBE) cross-sectional checklist when writing this article [23].

## 3. Results

### 3.1. Sample Characteristic

A total of 567 respondents was included in the analysis sample. Their socio-demographic characteristic in total sample, by professional groups, is presented in Table 1. As shown in this table, the professional groups by socio-demographic characteristics were not homogeneous. The groups of dental hygienists and dental assistants consisted exclusively of women. Dental hygienists differed from the other working groups by their younger age. As expected, the group of students was the youngest overall, and their work experience was limited to clinical practice undertaken during their studies.

### 3.2. Reasons for Child Neglect and Abuse

**Figure 1:**
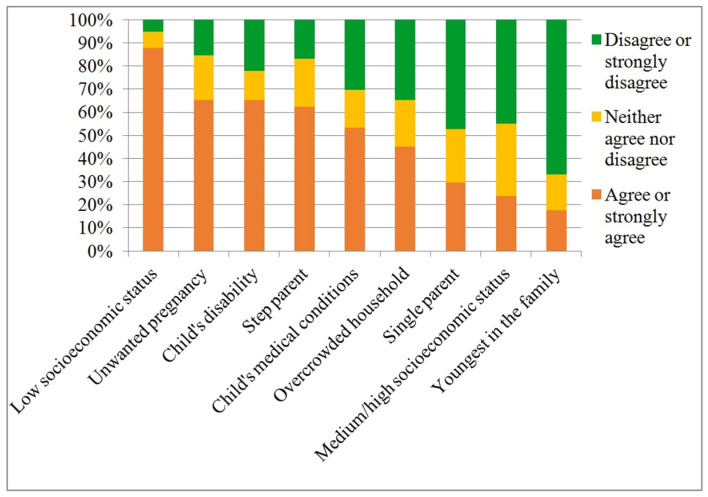
Distribution (%) of respondents’ answers to the question: “Do you think these factors may contribute to child abuse and neglect in the family?”.

Figure 1 presents respondents’ opinion on the reasons for child experience abuse and neglect in the family.

Of the nine listed reasons, the respondents mostly (87.7%) agreed with the statement that low socioeconomic status of the family is the reason for child abuse. Meanwhile, 23.6% of respondents agreed that average/high socioeconomic status of the family could also be a risk factor for child abuse and neglect, although many (31.4%) respondents had no opinion about this risk factor. A large proportion of respondents agreed with the statements that child abuse is related to the child’s disability (65.3%) and medical conditions (53.2%).

The listed child abuse-related reasons appeared to cluster into two distinct groups. To examine these groups, a factor analysis was conducted on nine items, which were well-suited for such analysis (KMO = 0.806; Bartlett’s test of sphericity, χ^2^(36) = 1710.87, *p* < 0.001). Two components with eigenvalues greater than one were extracted, together accounting for 54.6% of the total variance. Extraction communalities ranged from 0.304 to 0.835, indicating that most items were adequately represented by the factor solution.

Factor 1A reflected “Family social vulnerability”, capturing structural and socioeconomic conditions associated with the increased risk of child abuse. This factor showed strong positive loadings on low socioeconomic status of the family, family with step-parent, overcrowded household, unwanted pregnancy, and the child having a single parent. Sensitivity analysis indicated that the scores of this factor were not associated with gender (*B* = 0.189, *p* = 0.131) but were significantly associated with age (*B* = 0.370, *p* < 0.001).

Factor 2A represented “Child health-related vulnerability” and showed strong loadings on the presence of a medical condition and a physical or mental disability. Other items, such as being the youngest child in the family and medium/high socioeconomic status of the family, demonstrated weaker associations with this factor, as reflected by their lower communalities, indicating that they were less strongly explained by the extracted factors. Scores of this factor were not significantly related to neither respondents’ gender (*B* = 0.161, *p* = 0.204) nor age group (*B* = 0.086, *p* = 0.398); therefore, no adjustment for these characteristics was required in subsequent analyses of this factor.

The two factors were moderately negatively correlated (*r* = −0.482). This suggests that respondents who strongly endorsed family and social vulnerability factors tended to place relatively less emphasis on child health-related vulnerability, and vice versa.

Table 2 shows the average scores of these factors across the professional groups of dentists.

It was observed that there is a very small effect of professional specialization on Factor 1A (*F*(561, 4) = 1.880, *p* = 0.112, η^2^ = 0.013). However, pairwise comparisons using the LSD test indicated several significant differences in reporting family social vulnerability-related reasons for child abuse and neglect. For instance, this was more specific for dental assistants than for students (*p* = 0.037) or for dental specialists (*p* = 0.110). General dentists were also more likely to agree with these causes than students (*p* = 0.029). Significantly (*p* < 0.001) higher factor scores were observed among younger respondents (<40 years) compared with older respondents (≥40 years).

When analyzing Factor 2A, neither gender nor age was included in the model, as factor scores were not associated with these variables. Significant differences in Factor 2A scores across professional specialties were observed, *F*(562, 4) = 2.407, *p* = 0.048, although the effect size was small (η^2^ = 0.017). Dental assistants were the least likely to endorse child health-related reasons for child abuse and neglect. In contrast, dental specialists, dentists, and students were significantly more likely than dental assistants to agree that the risk of child abuse and neglect increases due to child health-related issues (*p* = 0.025, *p* = 0.032, and *p* = 0.011, respectively). These findings indicate that there is no consensus among dental professional groups about the causes of child abuse and neglect.

### 3.3. Referring of Child Abuse and Neglect

Our study also examined dentists’ opinions regarding when suspected cases of child abuse or neglect should be discussed or reported. Of the 553 respondents who answered the question, “When do you think a dentist should report cases of child abuse or violence against children?”, 419 (75.6%) respondents indicated that reporting is necessary “in all circumstances, even when abuse is only suspected”. This response option was selected most frequently by dentist specialists (83.7%) and least frequently by dental hygienists (63.6%), with the difference between professional groups reaching statistical significance (*p* = 0.009). The remaining respondents indicated that reporting should occur “in cases where violence is repeated” (11.4%), “only in cases of severe physical violence” (5.6%), or reported having “no opinion” (7.4%). Almost none of the respondents (one case) selected the option “never”, which was included in the questionnaire.

In cases of suspected child abuse or neglect, most respondents (67.0%) indicated that they would prefer to discuss their concerns with social services (State Child Protection and Adoption Service). Across professional groups, the largest difference in this preference was observed between dental specialists and dental hygienists (75.5% vs. 59.7%), although this difference did not reach statistical significance (*p* = 0.059). Other response options included discussing the concern with parents or family members (8.7%), reporting directly to the police (14.8%), and having no opinion (9.5%).

Additionally, we examined barriers to referring suspected cases to social services. To this end, a principal axis factor analysis with oblimin rotation was conducted on nine items assessing dentists’ perceived barriers to reporting child abuse and neglect. The Kaiser–Meyer–Olkin (KMO) measure indicated adequate sampling adequacy (KMO = 0.783), and Bartlett’s test of sphericity was significant, χ^2^(36) = 1071.53, *p* < 0.001. Two factors with eigenvalues greater than one were extracted, accounting for approximately 35% of the total variance. The pattern matrix, including the factor loadings of the scale items, is presented in Table 3.

The first factor, Factor 1B, labelled “Fear of negative consequences”, reflected concerns related to potential harm to the child or family, legal repercussions, and personal safety. The second factor, Factor 2B, labelled “Professional uncertainty”, captured issues related to limited diagnostic confidence, insufficient knowledge about referral procedures, and concerns regarding confidentiality. Several items—specifically fear of litigation, fear of negative effects on the child’s family, and confidentiality concerns—showed cross-loadings on both factors. The two factors were positively correlated (*r* = 0.609), indicating that dentists who experience greater fear of negative consequences also tend to report higher levels of professional uncertainty. Despite the presence of cross-loading items, the factor analytic approach substantially facilitated the analysis of perceived barriers by reducing the original nine items to two meaningful underlying dimensions measured with standardized factor scores.

A one-way analysis of variance (ANOVA) was conducted to examine differences in scores of fear of negative consequences (Factor 1B) and professional uncertainty (Factor 2B) across five professional groups: dentists, dental specialists, dental hygienists, dental assistants, and students (Table 4). Data in this analysis were adjusted for age groups, as both factors were sensitive to age (*B* = 0.512, *p* < 0.001, and *B* = 0.424, *p* < 0.001, respectively, for Factor 1B and Factor 2B). Sensitivity analysis also indicated that the expressions of these factors were not associated with gender (*B* = 0.070, *p* = 0.541, and *B* = 0.145, *p* = 0.155); therefore, no adjustment for age was required in subsequent analyses. The findings from ANOVA analysis suggested distinct professional profiles in relation to emotional and cognitive challenges in reporting about child abuse and neglect.

For Factor 1B, the analysis revealed a statistically significant effect of professional specialization *F*(561, 4) = 2.763, *p* = 0.027, with a small effect size (η^2^ = 0.019). Dental specialists reported the highest fear levels; pairwise comparisons using the LSD test indicated that their reported levels were significantly higher compared to dentists (*p* = 0.014), and the students’ group (*p* = 0.017). Students reported the lowest fear levels; their reported levels were significantly lower compared to dental specialists (*p* = 0.017), and dental hygienists (*p* = 0.025). Dentists also showed low levels of fear; their reported fear levels were significantly lower than those of specialists (*p* = 0.014) and hygienists (*p* = 0.042).

For Factor 2B, significant group differences were also observed, *F*(561, 4) = 5.446, *p* < 0.001, with a small but meaningful effect size (η^2^ = 0.037). Dental specialists reported the highest levels of professional uncertainty, significantly higher than any other specialty group (compared to dentists, *p* = 0.009; assistants, *p* = 0.001; and students, *p* < 0.001), except hygienists (*p* = 0.057). In contrast, students reported the lowest levels of uncertainty, which were significantly lower than those of dentists (*p* = 0.008), dental specialists (*p* < 0.001), and dental hygienists (*p* = 0.009).

For both factors, the opinion of dentists assessing barriers to reporting child abuse was significantly related to dentists’ age. Significantly (*p* < 0.001) higher factor scores of fear of negative consequences and professional uncertainty were observed among younger respondents (<40 years) compared with older respondents (≥40 years).

In summary, the results indicate that dental specialists were characterized by a high fear of negative consequences and the greatest professional uncertainty when reporting suspected child abuse or violence to institutions, despite being the professional group most likely to report suspected cases of child abuse or neglect. In contrast, students report the fewest difficulties with respect to the emotional and cognitive challenges associated with reporting obligations.

### 3.4. Knowledge

**Figure 2:**
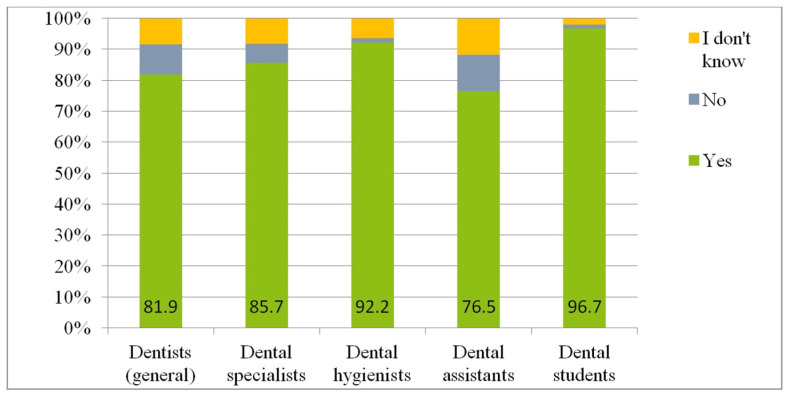
Distribution (%) of respondents’ answers to the question “Do you think you need more knowledge about child abuse and neglect?”, by professional specialization.

Only 14.8% of respondents reported having learned about child abuse and neglect during their studies, and a similar proportion (14.5%), therefore, perceived their knowledge on this topic as sufficient. Consequently, most participants (87.8%) supported the idea that child protection issues should be part of the undergraduate dental curricula, and 87.1% expressed a need for further training on this topic. Among all study participants, the need for more knowledge about child abuse and neglect was most frequently expressed by dental students, and least frequently by dental assistants (96.7% vs. 76.5%, *p* = 0.001) (Figure 2).

To assess respondents’ objective knowledge, participants were asked to answer 15 questions requiring selection of the correct response (“yes”, “no”, or “I don’t know”). The total number of correct responses was used as an indicator of knowledge level. Scores ranged from 1 to 15 (*M* = 10.53, SD = 2.80; median = 11). In sensitivity analyses, higher knowledge levels were observed among female compared to male respondents (*B* = 0.524, *p* = 0.134) and among younger (<40 years) compared to older (≥40 years) respondents (*B* = 0.448, *p* = 0.094); however, these differences did not reach statistical significance. Consequently, gender and age were not included in the ANOVA.

Knowledge levels differed significantly across professional groups, *F*(558, 4) = 4.342, *p* = 0.002, indicating that professional specialization explains about 3.0% of the variance in knowledge scores (Table 5). Dental specialists demonstrated the highest level of knowledge, while dental hygienists demonstrated the lowest level of knowledge (difference in mean of sum score between these specialties was significant, *p* = 0.014). Students’ knowledge levels were comparable to those of dental specialists.

However, the differences in findings among study participants may not reflect the significant differences in application of knowledge and attitudes in clinical practice.

## 4. Discussion

The present study described the knowledge and attitudes of employed dentists, dental specialists, assistants, hygienists and dental students towards child abuse and neglect in their professional practice and studies.

The first objective of this study was to explore dentists’ perceptions of reasons for child abuse within the family context. The findings indicate that most respondents perceived low family socioeconomic status, child disability, and medical conditions as important risk factors for child abuse and neglect. These perceptions are in line with the existing literature, where socioeconomic disadvantage and caregiver stress related to caring for children with disabilities or chronic health conditions are key risk factors for child maltreatment [1,2,3].

Socioeconomic adversity, including poverty and unemployment, has been repeatedly reported as one of the strongest predictors of child abuse and neglect [24,25]. Economic difficulties may affect parents’ capacity to provide adequate care by increasing exposure to stressors and reducing access to material and emotional resources, thereby heightening the risk of maltreatment [1]. The alignment between dentists’ perceptions and empirical evidence suggests an awareness among oral health professionals of the broader social determinants associated with child abuse and neglect.

The identification of child disability and medical conditions as risk factors is consistent with epidemiological evidence indicating that children with special healthcare needs are at increased risk of maltreatment [26,27]. Previous research has also demonstrated a significant association between child abuse and neglect and childhood disabilities [28,29]. Taken together, these findings underscore the importance of equipping dental professionals with the knowledge and skills necessary to recognize the increased vulnerability of children affected by socioeconomic disadvantage and health-related challenges.

Factor analysis of respondents’ opinions revealed that child abuse-related risk factors clustered into two dimensions: family social vulnerability and child health-related vulnerability. This finding is conceptually consistent with existing evidence, as children often experience multiple and overlapping forms of abuse and neglect throughout their lives. Previous research suggests that many risk factors for child abuse and neglect are shared across different social contexts, such as parental education level or ethnicity; however, relatively little attention has been paid to this issue and how these factors are perceived across professional groups [1].

In the present study, similarities in perceived reasons for child abuse were identified among dentists, who may be considered key professionals in the recognition of child maltreatment. Nevertheless, notable differences in attitudes across professional groups were also observed. Specifically, students and dental specialists were more likely than other professional groups to disagree with statements linking child abuse to family social vulnerability, while expressing stronger agreement that child abuse is associated with child disability and health-related factors. In contrast, dental assistants more frequently emphasized family social vulnerability over child health-related vulnerability.

These variations in perceptions across professional groups may reflect differences in education, clinical exposure, and professional roles, and they suggest the presence of gaps in training related to child maltreatment. Addressing these discrepancies through targeted undergraduate and continuing professional education may help promote a more comprehensive and consistent understanding of child abuse risk factors among oral health professionals.

The second objective of this study was to examine dentists’ attitudes regarding reporting about child abuse and neglect. Our study showed that three-quarters (75.6%) of respondents were convinced that dentists should report cases of child abuse or violence against children in all circumstances. This finding is in line with previous research showing that large proportions of dentists believe reporting suspected abuse is mandatory. For example, in a cross-sectional survey from India, conducted by Mahajan et al. [11], 97% of dentists felt that reporting suspected child abuse cases is mandatory, and 89% of respondents agreed that reporting cases of abuse is important, indicating broad agreement that dental practitioners should report such cases, as well as reinforcing that most dental professionals view reporting as a professional responsibility. Another study from India reported that almost 79% of dentists knew their legal duty to report suspected abuse, further supporting high levels of agreement on reporting responsibility. The latest study among Egyptian dentists found that about 82.2% of respondents believed that they play a significant role in perceiving and reporting child maltreatment cases, which demonstrates strong professional conviction about the importance of reporting [30].

We found that barriers to reporting child abuse can be clustered into dimensions of fear of negative consequences and professional uncertainty; however, these dimensions have different meanings across professional groups of dentists. This finding is consistent with previous research. A recent scoping review reported that diagnostic uncertainty, insufficient knowledge about reporting procedures, and the fear of repercussions for both the child and the practitioner are commonly pointed obstacles among dental health personnel [31]. In a Norwegian dental survey, barriers factored into lack of knowledge, fear of personal and clinic-related consequences, and fear for the child and family, with less trained professionals endorsing these barriers more strongly, suggesting variation across professional groups [32]. Additional studies have found that uncertainty about suspected abuse and the lack of clear reporting guidance reduce reporting behaviours, indicating that professional uncertainty remains a significant factor [33]. Likewise, surveys in other dental contexts have documented fear of misdiagnosis and fear of negative impact on the child or professional practice as important reporting barriers [34].

The third objective of this study was to assess dentists’ knowledge about child abuse and neglect. The knowledge test demonstrated that the surveyed dental personnel and dental students have good knowledge regarding child abuse and neglect, although a large majority of them stated not having enough knowledge. This finding is also consistent with previous research [30]. For example, a survey of dental professionals in India found that a substantial proportion of participants had good or fair knowledge and awareness of child maltreatment, yet many reported a lack of adequate knowledge and awareness about their professional role in identification and reporting [10]. Likewise, cross-sectional evidence from other study indicates that although many dentists can recognize signs of abuse, a large majority report insufficient training and uncertainty about how to act or whom to contact, which may limit reporting behaviours [35].

Overall, dental specialists and students demonstrated the highest knowledge levels, whereas dental hygienists demonstrated the lowest. This pattern suggests that advanced training (specialists) and recent education (students) may be associated with higher levels of up-to-date knowledge, whereas dental hygienists may have fewer formal opportunities or less curricular emphasis on this content area. These findings were identical to those reported in the study by Thomas et al. [36]. Finally, surveys of dental professionals have documented that knowledge scores differ significantly by educational qualification, suggesting that advanced training or higher academic exposure is associated with better understanding of child maltreatment issues [10].

The importance of protective factors that reduce the risk of child abuse and neglect has been increasingly recognized in the literature [1,27]. Oral health professionals, including dentists, may play an important role in strengthening such protective mechanisms through early identification, appropriate response, and referral. Based on the findings of this study and existing evidence, we seek to identify strategies that could support dentists in addressing this sensitive pediatric issue more effectively.

Our finding that many respondents still perceive their knowledge as insufficient underscores the need for optimizing education on child maltreatment. Consistent with previous research [10,34,35,36], our findings suggest that structured education on child abuse and neglect should be more comprehensively integrated into both undergraduate and postgraduate dental curricula. Such training should target all oral health professionals, including dental hygienists and assistants, who may have fewer formal educational opportunities in this area.

In addition, continuing professional development programmes focusing on legal responsibilities, the recognition of clinical and behavioural indicators, and reporting procedures may help to reduce the gap between knowledge and practice and decrease professional uncertainty. The development and dissemination of clear national or professional guidelines, together with decision-support tools, may further alleviate concerns about potential negative consequences by providing dentists with concrete and standardized procedural guidance. Furthermore, promoting interdisciplinary collaboration with other health professionals, teachers, and social workers, as well as establishing routine referral pathways to child protective services, could enhance dentists’ confidence and effectiveness in responding to suspected cases. At the practice level, the implementation of in-office policies, checklists, and documentation templates may support more consistent identification and reporting of child abuse and neglect [34,35,36]. Taken together, these measures—grounded on both the present findings and previous research—highlight actionable steps to strengthen the role of dental professionals in safeguarding vulnerable children.

However, several limitations of this study should be acknowledged. First, the questionnaire was limited in scope and did not cover several important aspects. For example, the list of potential causes of child abuse did not include sexual abuse, parental alcoholism or other parental mental health disorders, all of which are recognized risk factors. In addition, questions addressing the frequency of suspected maltreatment and the actions taken in response were not included. Such information would have provided valuable insights into clinical practice and helped identify ways to improve reporting rates. Future studies should therefore adopt a more comprehensive approach by broadening the scope of investigation. Second, the assessment of respondents’ knowledge was based on a limited number of theoretical statements requiring agreement or disagreement. This approach may not provide a sufficient accurate measure of knowledge, as it does not capture practical skills or decision-making in real-life situations. Furthermore, although respondents were instructed to provide their own answers, it is possible that some consulted with other study participants when completing the questionnaire, which may have introduced bias.

Other limitations are related to the study design. First, the study used a convenience sampling approach rather than a random sample. As participants were selected based on accessibility, the sample may not fully reflect the broader population, introducing potential selection bias. Consequently, the findings should be interpreted with caution and may not be generalizable to all members of the target population. Second, the study relies on self-reported data, which is subject to potential response bias. In particular, responses to sensitive questions, such as those concerning the reporting of child abuse or violence, may be influenced by social fear or social desirability bias. Efforts were made to minimize these effects by ensuring strict respondent anonymity.

## 5. Conclusions

The findings of this study highlight the critical importance of strengthening the knowledge and attitudes of oral health professionals, including students, in safeguarding children from abuse and neglect. Although Lithuanian dentists generally demonstrate moderate knowledge and attitudes in this area, the results indicate a clear need for more comprehensive and structured education at both undergraduate and postgraduate levels. Targeted training initiatives could enhance professionals’ confidence, diagnostic accuracy, and responsiveness when confronted with suspected cases of child maltreatment.

Future research should aim to address existing knowledge gaps, expand the scope of investigation across healthcare disciplines, and promote the development of standardized guidelines and reporting protocols to support timely, consistent, and effective child protection practices.

## Figures and Tables

**Table 1:** Sample characteristic.

Characteristic	Participants: *n*/Mean (%/SD)					
	All Specialties	Dentists (General)	Dental Specialists	Dental Hygienists	Dental Assistants	Dental Students
Total sample	567 (100)	237 (41.8)	49 (8.6)	77 (13.6)	51 (9.0)	153 (27.0)
Gender:						
Male	73 (12.9)	22 (9.3)	11 (22.4)			40 (26.1)
Female	494 (87.1)	215 (90.7)	38 (77.6)	77 (100)	51 (100)	113 (73.9)
Age (years):						
Mean (SD) ^1^	41.5 (13.5)	44.2 (14.0)	44.6 (12.3)	31.7 (7.0)	41.0 (12.8)	
<40 years	373 (65.3)	112 (47.3)	19 (38.8)	65 (84.4)	24 (47.1)	153 (100)
≥40 years	194 (34.2)	125 (52.7)	30 (61.2)	12 (15.6)	27 (52.9)	
Working experience:						
Mean (SD) ^1^	18.2 (13.1)	20.7 (13.8)	21.0 (13.3)	9.5 (7.3)	19.3 (13.6)	
<15 years	362 (63.8)	107 (45.1)	18 (36.7	62 (80.5)	22 (43.1)	153 (100)
≥15 years	205 (36.2)	130 (54.9)	31 (63.3)	15 (19.5)	29 (56.9)	
Employment status ^1^:						
Public sector	79 (19.1)	43 (18.1)	7 (14.3)	11 (14.3)	18 (35.3)	
Private sector	238 (57.5)	135 (57.0)	23 (46.9)	53 (68.8)	27 (52.9)	
Public and private	97 (23.4)	59 (24.9)	19 (38.8)	13 (16.9)	6 (11.8)	

Note: ^1^ estimated among employed respondents (*N* = 414).

**Table 2:** Descriptive statistics for factor scores assessing causes for child abuse and neglect according to the opinion of dentists by their professional specialization and age.

Group Definition	*n*	Factor 1A ^1^: *Family Social * * Vulnerability*		Factor 2A ^2^: *Child Health-Related * * Vulnerability*	
		*M*	(SE)	*M*	(SE)
Professional specialization:					
Dentists (general)	237	0.033	(0.064)	0.022	(0.065)
Dental specialists	49	−0.185	(0.142)	0.139	(0.142)
Dental hygienists	77	−0.079	(0.118)	−0.160	(0.113)
Dental assistants	51	0.132	(0.138)	−0.308	(0.139)
Dental students	153	−0.221	(0.095)	0.104	(0.080)
		*F*(561, 4) = 1.880		*F*(562, 4) = 2.407	
		*p* = 0.112		*p* = 0.048	
		η^2^ = 0.013		η^2^ = 0.017	
Age group:					
<40 years	373	0.128	(0.062)		
≥40 years	194	−0.256	(0.082)		
		*F*(561, 1) = 14.136			
		*p* < 0.001			
		η^2^ = 0.025			

Notes: ^1^ based on estimated marginal means; ^2^ based on raw data, as age group was not included in the model. Factor scores were standardized (overall *M* = 0), centred around zero; higher scores indicate greater endorsement of the respective factor. *M*: mean; SE: standard error; η^2^: effect size.

**Table 3:** Pattern matrix of the nine-item scale of barriers towards referring child abuse and neglect.

Items	Factor	
	1B *Fear of Negative Consequences*	2B *Professional * * Uncertainty*
Fear of family violence to the child	0.918	−0.210
Fear off consequences to the child	0.638	0.051
Fear of negative impact on dental practice	0.404	0.197
Fear of violence against dentist	0.398	0.040
Lack of certainty in diagnosis	−0.027	0.568
Lack of knowledge in referral procedures	−0.057	0.529
Concerns about confidentiality	0.207	0.482
Fear of litigation	0.342	0.346
Fear of negative effect on child’s family	0.312	0.316

**Table 4:** Descriptive statistics for factor scores assessing barriers to reporting child abuse according to opinion of dentists by their professional specialization and age.

Group Definition	*n*	Factor 1B ^1^: *Fear of Negative Consequences*		Factor 2B ^1^: *Professional * * Uncertainty*	
		*M*	(SE)	*M*	(SE)
Professional specialization:					
Dentists (general)	237	−0.137	(0.057)	−0.017	(0.051)
Dental specialists	49	0.201	(0.125)	0.309	(0.113)
Dental hygienists	77	0.107	(0.104)	0.025	(0.094)
Dental assistants	51	−0.056	(0.122)	−0.217	(0.111)
Dental students	153	−0.169	(0.084)	−0.264	(0.076)
		*F*(561, 4) = 2.763		*F*(561, 4) = 5.446	
		*p* = 0.027		*p* < 0.001	
		η^2^ = 0.019		η^2^ = 0.037	
Age group:					
<40 years	373	0.230	(0.055)	0.182	(0.050)
≥40 years	194	−0.251	(0.072)	−0.248	(0.065)
		*F*(561, 1) = 28.334		*F*(561, 1) = 27.740	
		*p* < 0.001		*p* < 0.001	
		η^2^ = 0.048		η^2^ = 0.047	

Notes: ^1^ based on estimated marginal means. Factor scores were standardized (overall *M* = 0), centred around zero; higher scores indicate greater endorsement of the respective factor. *M*: mean; SE: standard error; η^2^: effect size.

**Table 5:** Descriptive statistics assessing respondents’ knowledge about child abuse and neglect by dentists’ professional specialization.

Group Definition	*n*	Sum Score of Knowledge ^1^	
		*M*	(SE)
Professional specialization:			
Dentists (general)	237	10.31	(0.172)
Dental specialists	49	11.20	(0.380)
Dental hygienists	77	10.00	(0.300)
Dental assistants	51	10.48	(0.372)
Dental students	153	11.20	(0.213)
		*F*(558, 4) = 4.342	
		*p* = 0.002	
		η^2^ = 0.030	

Notes: ^1^ based on raw data. Higher sum scores indicate better knowledge. *M*: mean; SE: standard error.

## Data Availability

Data set available on request from the corresponding author.

## Supplementary Materials

The following supporting information can be downloaded at https://www.mdpi.com/article/10.3390/dj14060328/s1; File S1: Anonymous Questionnaire.
