# Supplementary material for: Knowledge and Attitudes of Dental Professionals in Lithuania Toward Child Abuse and Neglect: A Cross-Sectional Survey
Source: Dent J (Basel). 2026 Jun 1;14(6):328. doi: 10.3390/dj14060328 (PMC13297949; doi:10.3390/dj14060328)
Supplement: Supplementary file 1 [file dentistry-14-00328-s001.zip › dentistry-4244790-supplementary.pdf]

File S1

Anonymous Questionnaire

Hello. The aim of this work is to find out dental professionals and dental students' attitudes and assess knowledge about child abuse and neglect. We invite you to participate in the survey and answer the questions. This questionnaire is anonymous and all data collected will be summarized and used for scientific purposes only to write the master's thesis. Thank you for your time. If you have any questions, you can contact us by e-mail xx.xxx@lsmu.lt or by phone +370xxx xxxxx.

Example of marking: ☒ (choose one answer, except where it is written that multiple choice answers are possible).

1. Gender:
   - ☐ Female
   - ☐ Male

2. Age ………. years

3. Working experience ……….. years (for employers)

4. Employment status (for employers):
   - ☐ Public sector
   - ☐ Private sector
   - ☐ Public and private sectors

5. Professional specialization:
   - ☐ Dentist of general practice
   - ☐ Dental specialist (e.g., paediatric dentistry, oral surgery, orthodontics, periodontics, or prosthodontics)
   - ☐ Dental hygienist
   - ☐ Dental assistant
   - ☐ Dental student

6. Where do you study? (for students)
   - ☐ Lithuanian University of Health Sciences
   - ☐ Vilnius University

7. Choose the risk factors that you think influence the occurrence of child abuse and neglect. (*Mark the answer on each table line X*)

	Totally agree	Agree	Don't know	Disagree	Totally disagree
Child with disability (physical/mental disabilities)					
Child with medical condition					
Youngest in the family					
Unwanted pregnancy					
Family with step parent					
Child having single parent					
Overcrowded household					
Low socioeconomic status					
Medium/High socioeconomic status					

8. Choose symptoms / signs that you think may be related to physical child abuse. (*You can choose multiple answers*)
   - ☐ Labial/lingual fraenum tear
   - ☐ Laceration/burns to the lips
   - ☐ Fractures/dislocations/avulsions/pathologic mobility of teeth
   - ☐ Fracture of the condyle, ramus/symphysis of the mandible
   - ☐ Bite marks
   - ☐ Bruises behind the ears
   - ☐ Nasal fracture/clotted nostrils

9. Choose symptoms / signs that you think may be related to sexual abuse of a child. (*You can choose multiple answers*)
   - ☐ Erythema at the junction of the hard and soft palate
   - ☐ Fear of physical contact
   - ☐ Oral warts
   - ☐ Syphilis signs in the mouth
   - ☐ Herpes

10. Choose symptoms / signs that you think may be related to emotional / psychological child abuse. (*You can choose multiple answers*)
    - ☐ Pronounced nervousness
    - ☐ Self-inflicted injuries
    - ☐ Lack of self-esteem
    - ☐ Child tries to avoid any contact
    - ☐ Development delay
    - ☐ Being aggressive
    - ☐ Being passive

11. Choose symptoms / signs that you think may be related to the neglect of the child. (*You can choose multiple answers*)
    - ☐ Untreated rampant caries
    - ☐ Untreated pain, infection or trauma of the orofacial region
    - ☐ History of lack of continuity of care in the presence of identified pathology
    - ☐ Unattended medical needs
    - ☐ Poor personal hygiene

12. What should a dentist do first when he/she suspect child abuse? (*Choose one answer*)
    - ☐ Ask the child and parents about the signs/symptoms you noticed.
    - ☐ Document the signs/symptoms and your suspicion on the child‘s file
    - ☐ Monitor the case during the following visits
    - ☐ Discuss the case with colleague
    - ☐ Contact social services
    - ☐ Contact police
    - ☐ Check the consistency of parents/child explanation with the clinical findings
    - ☐ Do nothing
    - ☐ I don‘t know

13. In what circumstances do you think a dentist should report cases of child abuse? (*Choose one answer*)
    - ☐ All circumstance even if abuse is only suspected
    - ☐ In severe cases of physical abuse
    - ☐ In cases of where the physical violence to a child is repetitive
    - ☐ Never
    - ☐ I don‘t know

14. Who should be notified of suspected cases of child abuse? (*Choose one answer*)
    - ☐ Discuss the case with the child's family
    - ☐ Police
    - ☐ The State Child Rights Protection and Adoption Service
    - ☐ I don’t know

15. For what reasons do you think dentists usually do not report observed abuse and neglect of children? (*Mark the answer on each table line X*)

	Totally agree	Agree	Don‘t know	Disagree	Totally disagree
Fear of negative impact on dental practice					
Fear of family violence to the child					
Fear of violence against dentist					
Fear of litigation					
Fear off consequences to the child					
Lack of knowledge in referral procedures					
Lack of certainty in diagnosis					
Fear of negative effect on child‘s family					
Concerns about confidentiality					

16. Did you study about child abuse and neglect at university? (*If you answered no, go to question 14*)
    - ☐ Yes
    - ☐ No

17. What type of information on child abuse was provided? (*You can choose multiple answers*)
    - ☐ Theoretical information
    - ☐ Clinical information on signs and symptoms
    - ☐ Advanced clinical training in managing such cases
    - ☐ Information on how to report cases
    - ☐ Information on how to document cases

18. Do you think that the university should pay more attention to the issue of child abuse and neglect?
    - ☐ Yes
    - ☐ No
    - ☐ I Don’t know

19. Do you think you have sufficient knowledge to diagnose / suspect child abuse and neglect?
    - ☐ Yes
    - ☐ No
    - ☐ I Don’t know

20. Do you think you need more knowledge about child abuse and neglect?
    - □ Yes
    - □ No
    - □ I Don’t know

21. Deny or confirm statements based on knowledge about child abuse and neglect. (*Mark the answer on each table line X*)

	Agree	Disagree	Don’t know
Bruises on the cheek may indicate slapping or grabbing of the face			
Repeated injury to the dentition resulting in avulsed teeth or discoloured teeth may indicate repeated trauma from abuse			
Bruises noted around the neck are usually associated with accidental trauma			
Child abuse is primarily associated with the stresses of poverty and rarely occur amongst middle or high-income earners			
Children who have been abused usually tell someone soon after the abuse			
Child abuse may be indicated if a parent describes a child’s injury as a self-inflicted injury			
Child abuse may be indicated if a parent reports a child’s injury as a sibling inflicted injury			
Child abuse may be indicated if a parent delays seeking medical attention for a child’s injury			
Child abuse prevalence is less than Down syndrome prevalence			
Dentists can detect child abuse during their clinical practice			
Additional bruises usually occur in areas overlying bony prominences in abuse victims			
The abuser in most cases is someone the child knows well			
The best way to deal with suspected cases of child abuse is to confront the parents and accuse them directly of the abuse			
Emotional and psychological signs of abuse may include fear of going home or of the parents			
A history that is vague and differs every time the parent tells it is a possible indicator of abuse			

22. 5 years old patient, with a history of missing scheduled appointments, whose parents have already cancelled their visits several times, even though the need for treatment was reported during the first visits. When you look at the patient, you notice a worsening of the situation, early childhood caries, a lot of plaque, bleeding gums.

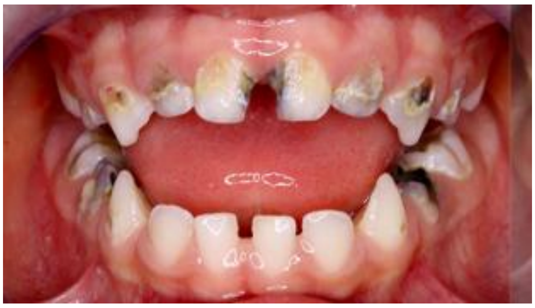

   a. Could child abuse be suspected in this case?
      - ☐ Yes
      - ☐ No
      - ☐ I Don't know
   b. If yes, what type of violence would you suspect?
      - ☐ Physical abuse
      - ☐ Sexual abuse
      - ☐ Psychological abuse
      - ☐ Neglect

23. Three years old child comes to your office and during the examination you notice an auricular hematoma of the outer ear. After the conversation with child mother about this injury, firstly she got confused, but immediately replied that the child had slipped up the stairs and injured himself.

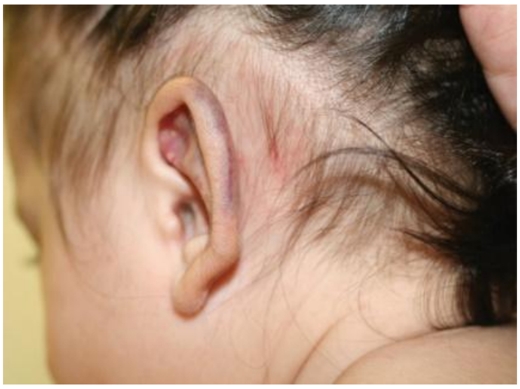

   ☐ Could child abuse be suspected in this case?
      - ☐ Yes
      - ☐ No
      - ☐ I Don't know
   b. If yes, what type of violence would you suspect?
      - ☐ Physical abuse
      - ☐ Sexual abuse
      - ☐ Psychological abuse
      - ☐ Neglect
